# Seeing the Unseen: Enhanced Stent Visualization Reveals Hidden Coronary Stent Complications

**DOI:** 10.3390/jcm15103907

**Published:** 2026-05-19

**Authors:** Carlotta Rossignoli, Chiara Bianchi, Hesham Abu Abied, Alberto Zamboni, Francesco Bacchion, Giorgio Morando, Antonio Mugnolo, Simone Biscaglia, Gabriele Venturi

**Affiliations:** 1 Department of Cardiology, Azienda Ospedaliera Universitaria Integrata di Verona, 37126 Verona, Italy; rossignolicarlotta@gmail.com (C.R.);; 2 Department of Cardiology, Università degli Studi di Ferrara, 44121 Ferrara, Italy; chiarabianchi3496@gmail.com (C.B.);; 3 Department of Cardiology, Ospedale Mater Salutis di Legnago (VR), 37045 Legnago, Italy

**Keywords:** enhanced stent visualization (ESV), percutaneous coronary intervention (PCI), stent-related complications, complex coronary lesions, intravascular imaging

## Abstract

**Background**: Accurate evaluation of stent implantation during percutaneous coronary intervention (PCI) is essential to reduce both early and late adverse events. Conventional coronary angiography, although routinely used, has limited spatial resolution and may fail to detect subtle mechanical abnormalities in implanted stents. Enhanced stent visualization (ESV) is an X-ray-based post-processing technique that improves delineation of stent struts without additional contrast or intracoronary instrumentation. **Methods**: We report a retrospective case series of five patients who underwent complex PCI where ESV was used as an adjunctive imaging modality. Clinical scenarios included left main interventions, bifurcation lesions, multivessel disease, and acute coronary syndromes. The ability of ESV to detect mechanical complications not evident on angiography was assessed. The impact of ESV on procedural decision-making was also assessed. **Results**: ESV enabled identification of mechanical complications in all cases, including stent fracture, stent loss, stent dislodgement, stent underexpansion, and geographical miss. These findings were not clearly appreciable when using angiography alone. In each case, ESV directly influenced intraprocedural management, prompting immediate corrective actions such as additional stent implantation, stent retrieval, or further optimization with post-dilatation or intravascular lithotripsy. This resulted in improved procedural outcomes and optimized stent deployment. **Conclusions**: In this small retrospective case series, ESV provided incremental diagnostic value over conventional angiography by detecting otherwise unrecognized mechanical complications and guiding real-time procedural optimization. While these findings suggest a potential role for ESV in complex PCIs, larger prospective studies are required to confirm its clinical impact.

## 1. Introduction

Optimal assessment of procedural results after percutaneous coronary intervention (PCI) is essential to reduce acute and long-term complications, including in-stent restenosis and stent thrombosis. Although conventional coronary angiography remains the cornerstone for intraprocedural evaluation, it has well-recognized limitations in accurately visualizing stent architecture, particularly with respect to stent failure. More specifically, stent failure is a multifactorial phenomenon mainly driven by mechanical complications intended as specific abnormalities related to the physical integrity, position, expansion, or spatial coverage of the implanted coronary stent. Indeed, mechanical complications refer to stent underexpansion, malapposition, fracture and edge disease, in contrast with thrombotic, biological, or pharmacological causes of stent failure.

To overcome these limitations, enhanced stent visualization (ESV), an X-ray-based imaging technology, has been developed, allowing improved delineation of stent struts through post-processing of standard fluoroscopic images. The two major ESV tools are StentBoost (Philips Medical Systems Nederland B.V., Best, The Netherlands, https://acc.healthcare.philips.com/healthcare/product/HCSBL02/stentboost-live-see-clearly-stent-confidently, accessed on 14 May 2026) and ClearStent (Siemens Healthineers, Erlangen, Germany, https://www.siemens-healthineers.com/angio/options-and-upgrades/clinical-software-applications/clearstent, accessed on 14 May 2026). These are widely available, easy to implement in the catheterization laboratory, and do not require additional intravascular instrumentation or contrast administration. By enhancing the radiopacity of metallic stent structures, ESV enables more precise assessment of stent geometry immediately after deployment. Indeed, it can lead to improvement in intraprocedural decision-making, such as additional post-dilatation or optimization strategies. Furthermore, a correlation has been reported between ESV findings and intravascular imaging modalities, including intravascular ultrasound (IVUS) and optical coherence tomography (OCT), particularly for the detection of stent underexpansion and malapposition. More specifically, IVUS and OCT provide cross-sectional, high-resolution tomographic imaging: the first is better for the assessment of vessel size and stent underexpansion, especially in calcified or large vessels; the second offers higher spatial resolution and allows a more precise detection of malapposition and edge dissections [1,2,3]. However, ESV correlated with IVUS and OCT for gross stent expansion and positioning, but IVUS and OCT remain the gold standard for the assessment of stent deployment and prediction of clinical outcomes during PCI [1,3,4,5].

ESV is preferred in patients with complex coronary lesions such as bifurcations, left main (LM) disease, long lesions, severe calcification, or chronic total occlusion, and in those presenting with acute coronary syndromes (ACSs), including ST-elevation myocardial infarction (STEMI), non-ST-elevation myocardial infarction (NSTEMI) and unstable angina. The American College of Cardiology, American Heart Association, and collaborating societies recommend the use of intracoronary imaging for optimization of stent deployment in ACS and PCI for complex lesions, confirming that these patient populations derive the greatest clinical benefit from improved procedural outcomes and reduced adverse events [6]. IVUS and OCT remain the guideline-supported imaging modalities, but ESV should be considered an adjunctive angiographic enhancement tool that may provide additional information on stent geometry, although it cannot replace intravascular imaging. Short- and long-term studies have investigated the impact of ESV-guided PCI on clinical outcomes. In the studies by Duan et al. and Oh et al., no significant differences in short-term outcome were proven, but long-term outcomes at 12 months demonstrated a significant reduction in adverse events, with lower rates of target lesion restenosis (TLR) and TLR-MACE. Indeed, immediate clinical benefits may not be gained with ESV-guided PCI, but long-term outcomes may be achieved in terms of reduced revascularization needs and MACE rates [7,8].

In this context, we present a retrospective case series of five patients in whom the use of ESV allowed clear identification of stent fracture, stent loss, stent dislodgement, stent underexpansion, and geographical miss, none of which were evident on standard angiography. The aim of this case series is to illustrate the incremental value of ESV in identifying stent-related complications and to describe its impact on intraprocedural decision-making in complex PCI scenarios. While previous studies have evaluated the impact of ESV-guided PCI on clinical outcomes at a population level, our case series provides a complementary, practice-oriented perspective by illustrating how ESV can influence real-time procedural decision-making.

We want to underline that, being a case series, a limited number of cases is evaluated, and the study is primarily descriptive and illustrative. Moreover, we deliberately selected a one-year time window for case inclusion; this allowed us not only to collect representative and consecutive cases but also to ensure the availability of a 12-month clinical follow-up (outpatient visits at 1 and 12 months). Within this period, no procedure-related complications or adverse events were observed, supporting the safety and appropriateness of the ESV-guided management strategies described.

## 2. Clinical Cases

### 2.1. First Scenario: Stent Fracture

**Figure 1:**
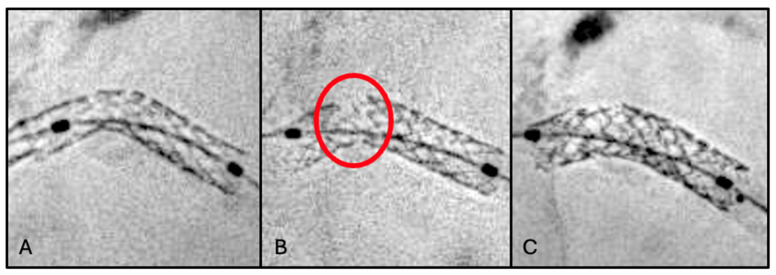
(**A**) Optimal stent positioning to LM-LCx bifurcation during primary PCI (first procedure). (**B**) Stent fracture (red circle) clearly visualized using StentBoost enhancement at second procedure. (**C**) Successful bailout treatment with in-stent implantation, achieving an adequate final angiographic result.

A 75-year-old man with chronic coronary syndrome was admitted for unstable angina. Cardiovascular risk factors were hypertension and dyslipidemia (baseline LDL cholesterol was 118 mg/dL). Previous cardiovascular history was significant for coronary artery bypass grafting (CABG) with arterial graft to the left anterior descending artery (LAD), PCI with two drug-eluting stents (DESs) to the right coronary artery (RCA), and PCI with DES implantation to the left main–left circumflex (LM-LCx) bifurcation (Figure 1A) 3 years previously. Home therapy included aspirin, statin therapy (atorvastatin 40 mg), and beta-blockers.

Coronary angiography at index procedure revealed severe in-stent restenosis of the LM-LCx, moderate disease progression in the RCA, and a patent arterial graft to the LAD. StentBoost imaging demonstrated proximal stent fracture associated with significant stent underexpansion in the LM-LCx segment (Figure 1B). Stent fracture probably led to loss of structural integrity and focal mechanical instability, resulting in areas of altered vessel scaffolding and abnormal stress distribution; this could have promoted neointimal hyperplasia and contributed to in-stent restenosis, particularly in segments exposed to repetitive mechanical strain such as the LM-LCx bifurcation.

Clarifying the mechanism of stent restenosis, an in-stent stenting was adopted as therapy. PCI of the LM-LCx was performed, with implantation of one DES (3.0 mm × 16 mm) (Figure 1C). Final IVUS confirmed adequate stent expansion and apposition, complete coverage of the diseased segment, and restoration of TIMI grade 3 flow.

### 2.2. Second Scenario: Stent Loss

A 56-year-old man was scheduled for LM angioplasty as a staged procedure following STEMI caused by RCA occlusion. He was an active smoker with chronic ischemic heart disease, hypertension and dyslipidemia (LDL cholesterol was 132 mg/dL at admission). Medical therapy included aspirin, ticagrelor, statin (rosuvastatin 20 mg), ACE inhibitor, and beta-blocker.

The first step involved PCI of the LCx, with implantation of one DES (3.0 mm × 16 mm). This was followed by PCI of the LM-LAD, with implantation of one DES (3.5 mm × 32 mm).

**Figure 2:**
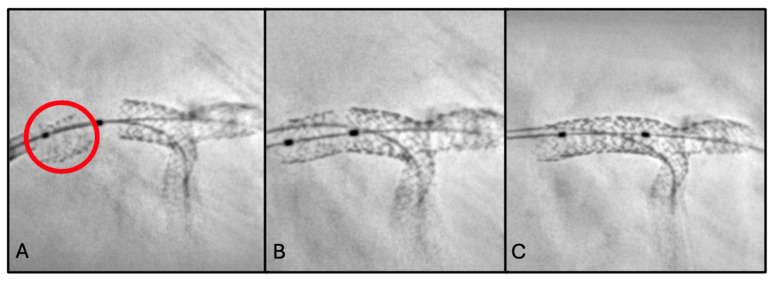
(**A**) StentBoost-enhanced visualization of stent loss in the LM (red circle). (**B**) Crossing of the dislodged stent with two balloons followed by balloon inflation to secure adequate anchoring. (**C**) Final angiographic evaluation with StentBoost, confirming maintenance of optimal bifurcation geometry and appropriate stent apposition.

Post-deployment angiography revealed acute occlusion of the LCx ostium secondary to plaque shift. Following successful rewiring and predilatation, forceful advancement of the stent was required to achieve LCx stent delivery, owing to unfavorable bifurcation anatomy and severe calcific disease. StentBoost imaging revealed loss of an unexpanded stent within the LM (Figure 2A). The lost stent was sequentially crossed with two balloons (0.75 mm and 1.25 mm) and expanded to achieve anchoring (Figure 2B). Wire, guiding catheter, and stent were then withdrawn en bloc up to the humeral artery. During retrieval, distal embolization of the stent to the ulnar artery occurred, without angiographic evidence of flow limitation or ischemic sequelae.

To complete the procedure, right femoral access was obtained. The LCx was successfully re-crossed, and PCI was performed with DES implantation (3.0 mm × 8 mm) using the T and Protrusion (TAP) bifurcation technique. Final kissing balloon inflation was performed to optimize bifurcation geometry and stent apposition. IVUS confirmed adequate stent expansion, apposition, and coverage of both the LCx ostium and the LM-LAD segment. Final angiographic result was optimal, with restoration of TIMI grade 3 flow in all treated vessels (Figure 2C).

To be more precise, in this case “stent loss” refers to unintentional complete loss of the stent from the delivery system, with consequent migration to a location outside the intended coronary segment before deployment.

### 2.3. Third Scenario: Stent Dislodgement

A 70-year-old man was admitted for NSTEMI. The patient had diabetes mellitus, hypertension, and dyslipidemia (LDL cholesterol 124 mg/dL). Home therapy included aspirin, clopidogrel, statin therapy, and glucose-lowering medications.

Coronary angiography demonstrated severe diffuse disease of the RCA, which was identified as the culprit vessel. The lesion was treated with primary PCI consisting of drug-coated balloon (DCB) angioplasty and implantation of four DESs, achieving satisfactory angiographic results.

Residual coronary artery disease included borderline stenosis of the distal LM, severe ostial stenosis of the LAD, and severe stenosis of the mid LCx. Given the anatomical complexity, a staged PCI approach was planned.

During the second procedure, PCI of the LCx was performed, with implantation of one DES (2.75 mm × 20 mm) in the mid segment. Subsequently, the ostial-proximal LAD was treated with balloon angioplasty followed by implantation of one DES (3.5 mm × 16 mm) from the LM into the LAD.

Post-deployment angiographic assessment showed plaque shift involving the proximal LM segment. Consequently, an additional DES (4.5 mm × 8 mm) was implanted in the LM to ensure adequate lesion coverage and stent apposition.

**Figure 3:**
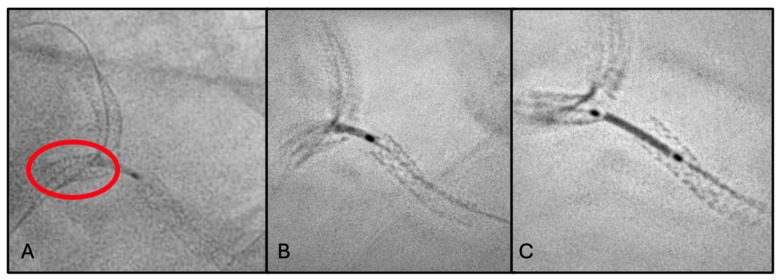
(**A**) StentBoost-enhanced image showing distal LM stent (red circle). (**B**) Retrieval of the dislodged stent using the anchoring balloon technique. (**C**) StentBoost confirmation of optimal stent expansion, proper apposition, and preservation of LM bifurcation geometry.

Final ESV unexpectedly revealed distal stent dislodgement, with partial protrusion and floating of the stent between the LM and the ascending aorta (Figure 3A). A percutaneous stent retrieval maneuver was promptly undertaken using an anchoring balloon technique (5.0 mm × 8 mm), allowing successful repositioning of the dislodged stent within the LM (Figure 3B).

To secure the entire LM bifurcation and prevent further mechanical instability, an additional DES (4.0 mm × 12 mm) was implanted to overlap and seal the previously deployed stents. Final angiographic control demonstrated optimal stent expansion, correct apposition, preserved LM bifurcation geometry, and restoration of TIMI 3 flow in all treated vessels (Figure 3C).

In this case “stent dislodgement” refers to partial displacement of the stent from its intended landing zone after deployment, while it still remains within the coronary circulation in proximity to the target vessel.

### 2.4. Fourth Scenario: Stent Underexpansion

A 63-year-old man with history of exertional chest pain was admitted for NSTEMI. He had hypertension and severe dyslipidemia (LDL cholesterol of 145 mg/dL). He was on aspirin and moderate-intensity statin therapy.

Coronary angiography showed a critical stenosis of the LM-LAD bifurcation and a critical stenosis of the mid-distal segment of the RCA.

**Figure 4:**
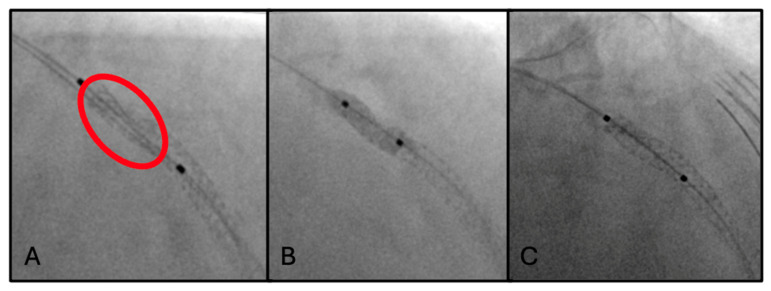
(**A**) ClearStent-enhanced visualization revealing proximal stent underexpansion (red circle) secondary to severe calcification of the LAD. (**B**) High-atmosphere balloon dilatation. (**C**) Final angiography confirming an optimal procedural outcome.

PCI of mid and proximal LAD was performed with implantation of two DESs (2.5 mm × 15 mm and 3.0 mm × 38 mm), achieving a good final angiographic result. However, ClearStent technology showed proximal stent underexpansion due to severe calcifications of the vessel (Figure 4A).

Optimization of the expansion was then achieved with the use of bailout Intravascular Lithotripsy (IVL) in-stent, followed by high-pressure post-dilations with non-compliant balloons of progressive caliber (Figure 4B). In addition, IVL was used for lesion preparation at the level of LM-LAD bifurcation, with subsequent further DES implantation (4.0 mm × 33 mm) overlapping the previous one proximally. A good final angiographic result (Figure 4C) was confirmed by an IVUS run, which demonstrated the achievement of a good Minimum Lumen Area (MLA) despite the persistence of a focal area of underexpansion.

### 2.5. Fifth Scenario: Missed Overlap

An 85-year-old man was admitted with an inferior STEMI. Cardiovascular risk factors included hypertension, dyslipidemia (LDL cholesterol 110 mg/dL), and a family history of ischemic heart disease. Home therapy included aspirin, clopidogrel, and statin.

Successful primary PCI of the RCA with DES implantation was performed.

Staged PCI on left coronary artery was planned during the same hospitalization. After wiring the LAD and the first diagonal branch (D1), two DESs (2.5 mm × 28 mm and 3.0 mm × 36 mm) were released on the middle and distal segments of LAD. A third DES (3.5 mm × 24 mm) was added on proximal LAD-D1 bifurcation. Then, after protective wiring of LCx, a fourth DES (4.0 mm × 28 mm) was added in overlap on the LM-proximal LAD bifurcation.

**Figure 5:**
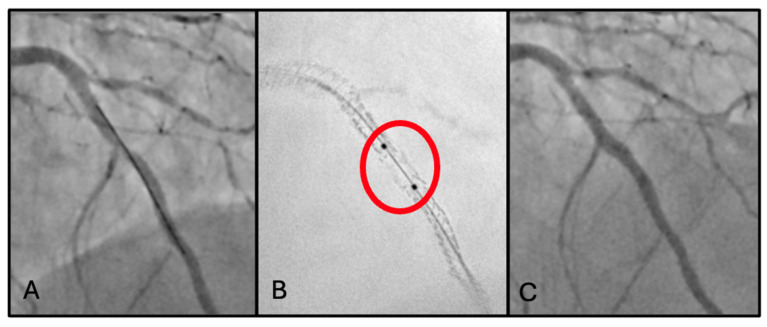
(**A**) Angiography showing a suboptimally visualized segment in the mid-distal LAD following stent implantation. (**B**) ClearStent-enhanced imaging identifying a gap between the two previously implanted stents on the LAD (red circle). (**C**) Final angiography confirming an excellent result after placement of an additional stent to bridge the gap.

Angioplasty of the entire axis was then optimized with post-dilation using non-compliant balloons, while the double bifurcation stenting was finalized through the Proximal Optimization Technique (POT). However, the final angiography demonstrated a non-clearly optimized portion of the vessel in the mid-distal LAD (Figure 5A), which a ClearStent scan revealed to be a gap between the two stents due to a lack of overlap (Figure 5B).

The procedure was then completed with additional implantation of a fifth and final DES (3.0 mm × 12 mm) to cover the gap, achieving an excellent final angiographic result (Figure 5C).

## 3. Discussion

Overall, the accurate assessment of stent deployment is crucial for procedural success and optimal clinical outcomes in PCI. Coronary angiography, although routinely employed in clinical practice, has intrinsic limitations in the accurate assessment of stent deployment. Mansour et al. reported that PCI guided exclusively by angiography was associated with inadequate stent expansion in 38% of cases, emphasizing the need for more reliable imaging techniques during the procedure. ESV represents a valuable adjunct, as it improves the visibility of stent struts and margins through advanced digital processing of fluoroscopic images, allowing a more precise identification of underexpansion, suboptimal stent apposition, and stent fracture [9,10]. Consistently, Blicq et al. demonstrated that ESV was able to detect stent underexpansion in 18% of lesions that were judged satisfactory on angiography, confirming its higher diagnostic sensitivity compared with angiographic assessment alone [11].

In terms of evaluation of correct stent deployment, advanced intravascular imaging modalities such as OCT and IVUS are still considered the gold standards for evaluating stent expansion and integrity. These cannot be replaced by ESV; however, their routine use is often constrained by cost, by their limited availability globally, and by procedural complexity. ESV represents an imaging approach positioned between standard coronary angiography and intravascular imaging techniques. It offers improved diagnostic accuracy over angiography in assessment of stent expansion and procedural complications, while providing results that are largely comparable to those of intravascular imaging for the evaluation of stent sizing and optimization [12].

In our cases, when integrated with intravascular imaging, ESV findings were consistent with IVUS-confirmed optimization of stent expansion, apposition, and lesion coverage, supporting its role as a complementary rather than competitive modality. ESV may assist intraprocedural decision-making, mostly when intravascular imaging is not available or feasible. On the one hand, ESV provides improved stent visualization compared with angiography alone; on the other hand, IVUS allows high-resolution imaging and achieves improved clinical outcomes, as already demonstrated in the literature. Indeed, reliance on ESV alone risks missing clinically significant complications that IVUS can detect, potentially leading to worse clinical outcomes.

The main concern with ESV is radiation exposure and the risk of related complications. However, Fysal et al. showed only a 3.7% increase in radiation exposure in ESV-guided PCI [13]. Moreover, according to Jin et al., no significant difference in dose-area product, fluoroscopy time or cine frames was reported in ESV compared to non-ESV [14].

Overall, this experience reinforces the concept that ESV represents a promising imaging adjunct that bridges the gap between conventional angiography and intravascular imaging, enabling a more mechanically optimized, more individualized and safer PCI, particularly in cases where stent failure mechanisms are subtle or angiographically occult.

## 4. Limitations

Our study has some limitations inherent to its design as a retrospective case series. The small sample size and absence of a control group limit the generalizability of our findings. In addition, ESV was not systematically compared with gold-standard intravascular imaging modalities such as IVUS or OCT in all cases. The analysis is primarily descriptive, and focuses on procedural aspects, without the ability to establish causal relationships or assess any impact on long-term clinical outcomes. Finally, although clinical follow-up at 1 and 12 months was available, and was uneventful in all patients, no systematic imaging follow-up was performed.

## 5. Future Perspectives

Based on our observations, ESV may be increasingly considered as a routine or selective intraprocedural screening tool, particularly in complex anatomical settings such as bifurcation lesions, heavily calcified segments, LM interventions, in-stent restenosis, and multi-stent implantations. Additionally, ESV may play a growing role in ultra-low-contrast or zero-contrast PCI, especially in patients with advanced chronic kidney disease, by reducing reliance on contrast angiography while maintaining procedural safety and efficacy [15]. Wide availability, ease of use, a lack of need for additional contrast administration, and a minimal impact on procedural time all make ESV an attractive adjunct in daily catheterization laboratory practice, so that ESV may be expected to evolve from a standalone angiographic enhancement tool to a complementary modality that can be fused with intravascular imaging and physiological data [3,5,15,16]. However, it is important to remember that ESV provides a potential supportive role, without implying equivalence with guideline-recommended intravascular imaging techniques.

Future prospective studies involving large patient cohorts are warranted to strengthen systematic or targeted use of ESV and corroborate its association with a reduction in short- and long-term adverse events. Moreover, further research may enhance the diagnostic performance, reproducibility, and standardization of ESV techniques.

## 6. Conclusions

In the presented cases, ESV enabled prompt identification of mechanical complications which were not clearly appreciable on angiography despite careful evaluation. Importantly, the additional information provided by ESV had a direct and immediate impact on procedural decision-making, guiding bailout strategies and leading to immediate corrective actions including further stent implantation, post-dilatation, and stent retrieval techniques. Therefore, the clinical impact of ESV was mainly assessed in terms of real-time procedural decision-making rather than long-term outcomes. However, clinical follow-ups at one and twelve months with an absence of symptoms or hospitalizations (for cardiac reasons) corroborate the successful procedural results.

In the first scenario, StentBoost allowed precise identification of stent fracture and further significant stent underexpansion, neither of which could be adequately assessed by angiography alone. By improving stent visualization, StentBoost provided essential information to support appropriate decision-making and guide the ideal bail-out strategy. The complementary use of IVUS further confirmed the optimal PCI outcome.

The procedure illustrated in the second scenario was intrinsically high-risk due to the recent STEMI, the multivessel coronary artery disease, the LM involvement, and the unfavorable bifurcation anatomy, factors all associated with increased procedural complexity and potentially severe complications. StentBoost enabled immediate recognition of stent loss and facilitated real-time decision-making during a critical phase of the procedure. Indeed, the lost stent was not clearly identifiable on conventional angiography, likely due to its unexpanded state, low radiographic profile, and overlap with other metallic structures in a complex LM bifurcation setting. ESV enhanced the visualization of metallic struts, allowing clear identification of the unexpanded stent and enabling appropriate management.

The third clinical case confirmed the crucial role of StentBoost in the setting of a complex PCI. It promptly detected the stent dislodgement and guided the rescue maneuvers, significantly improving procedural safety.

In the fourth scenario, the key role of ClearStent was demonstrated by the detection of stent underexpansion, a critical mechanical issue that would likely have remained unrecognized with angiography alone. The optimization of procedural results was later confirmed by IVUS, enhancing again the synergistic work of these two imaging modalities.

In the final case, despite extensive stenting and an apparently acceptable angiographic result, conventional fluoroscopy was unable to clearly identify a geographical miss caused by an unrecognized gap between two stents. ClearStent allowed prompt detection of the complication, confirming its valuable adjunctive role to angiography.

In our experience, ESV did not significantly lengthen procedural time nor substantially increase complexity. Any increase in procedural duration was minimal and mainly related to image acquisition and interpretation, both of which were rapidly integrated into routine workflow.

## Data Availability

Data of this study are available from Ospedale Mater Salutis (Legnago) and Università degli Studi di Ferrara, but restrictions apply to availability of these data, which were used under license for the current study, and so are not publicly available (sensitive data).

## Abbreviations

The following abbreviations are used in this manuscript:

PCI	Percutaneous coronary intervention
ESV	Enhanced stent visualization
IVUS	Intravascular ultrasound
OCT	Optical coherence tomography
LM	Left main
ACS	Acute coronary syndrome
STEMI	ST-elevation myocardial infarction
NSTEMI	Non-ST-elevation myocardial infarction
TLR	Target lesion restenosis
CABG	Coronary artery bypass grafting
LAD	Left anterior descending artery
DES	Drug-eluting stent
RCA	Right coronary artery
LCx	Left circumflex artery
TAP	T and protrusion
DCB	Drug-coated balloon
IVL	Intravascular lithotripsy
MLA	Minimum lumen area
D1	Diagonal branch
POT	Proximal optimization technique
